# Evaluating the role of the self-assembling topical haemostat PuraBond® in Transoral Robotic Surgery (TORS) for oropharyngeal cancer: A case series

**DOI:** 10.1016/j.amsu.2022.104302

**Published:** 2022-08-01

**Authors:** Keshav Kumar Gupta, Georgios Garas, Matthew Idle, Susan Germain, Mriganka De

**Affiliations:** a Head & Neck Unit, University Hospitals Birmingham NHS Foundation Trust, Mindelsohn Way, Birmingham, B15 2BW, United Kingdom; b Speech & Language Therapy Department, Birmingham Heartlands Hospital, University Hospitals Birmingham NHS Foundation Trust, Bordesley Green East, Birmingham, B9 5SS, United Kingdom

**Keywords:** Transoral robotic surgery, Human papilloma virus, Oropharyngeal cancer, Haemostat, Case series, TORS, Transoral robotic surgery, HPV, Human papilloma virus

## Abstract

**Background:**

Transoral Robotic Surgery (TORS) has been increasingly employed in head and neck surgery for the assessment and treatment of malignancies over the last two decades. PuraBond® is a self-assembling viscous solution that forms a transparent hydrogel 3-D matrix to promote local haemostasis. This study aimed to assess the utility of PuraBond® in patients undergoing TORS for Human Papilloma Virus (HPV) positive oropharyngeal squamous cell carcinoma (OPSCC).

**Methods:**

All patients who underwent TORS with PuraBond® between October 2021–May 2022 at a single tertiary university hospital in the United Kingdom were included. Primary outcome measures included post-operative haemorrhage rate (primary; within 24hrs of surgery, secondary; 1–30 days post-surgery). Secondary outcome measures included, Length Of hospital Stay (LOS), swallowing complications, hospital re-admission, and surgeon-reported ease of PuraBond® application.

**Results:**

Twelve patients were included (13 procedures due to one second look and re-resection case). No patients developed primary or secondary post-operative haemorrhage. There were no re-attendances within 30 days. Average LOS was 2.78 days (range: 1.54–4.31 days). No patient required feeding tube insertion or tracheostomy. In all procedures, the use of PuraBond® was reported as ‘easy’.

**Conclusion:**

This is the first study to evaluate the role of PuraBond® in TORS. The wide range of favourable outcomes reported support its safety and efficacy. The current findings mandate the need for larger, prospective, controlled studies to better define whether the known haemostatic and regenerative properties of PuraBond® may translate into direct patient benefit in the expanding field of TORS for HPV-mediated OPSCC.

## Highlights

• Major haemorrhage is a key concern following transoral robotic surgery (TORS).
• Purabond® is a synthetic haemostatic agent used in other surgical settings.
• Our series is the first to evaluate the role of Purabond® in TORS.
• We demonstrate excellent outcomes in terms of bleeding and swallowing complications.
• Larger, prospective controlled studies are needed to further assess these outcomes.

## Introduction

Transoral Robotic Surgery (TORS) has been increasingly utilised in Ear, Nose and Throat (ENT) and Head and Neck Surgery over the past two decades offering unprecedented 3-dimensional (3-D) views and enabling access to traditionally ‘difficult-to-reach’ tumours. At the same time, robotic technology enhances surgical precision through wristed robotic instruments with seven degrees of freedom, tremor-filtering, and motion scaling [1]. The primary role of TORS in ENT is in the assessment and treatment of carcinoma of unknown primary (CUP) and head and neck malignancies in a variety of subsites, respectively [2]. Traditionally, these cancers were surgically managed necessitating major open approaches (e.g. involving mandibulotomy, lateral pharyngotomy) that carry substantial morbidity [3], thus resulting in chemoradiotherapy (CRT) eventually becoming the mainstay treatment [4].

The subsequent emergence of TORS swung the pendulum back towards surgery. TORS constitutes a viable primary treatment option, especially in the case of Human Papilloma Virus (HPV) positive early-stage oropharyngeal squamous cell carcinoma (OPSCC) where it has been shown to offer equivalent or superior oncological and functional outcomes (compared to CRT) in a variety of studies [[5], [6], [7], [8], [9], [10]]. At the same time, as with all surgical procedures, TORS is not without risk. The most feared complication of TORS is post-operative haemorrhage. Reported rates for this vary but range between 4.1 and 9.8% [[11], [12], [13]] though there have been concerns that this may be underreported [8]. Haemorrhage following TORS has the potential to be catastrophic through the combination of airway compromise and hypovolaemia, resulting in death [14].

To reduce haemorrhage across a variety of surgical settings, several haemostatic agents have been developed that employ an array of different mechanisms to promote topical haemostasis. An example of such agent is PuraBond® (also known as PuraStat®; 3D Matrix Ltd, Tokyo, Japan), which consists of the RADA16 family of synthetic peptides. PuraBond® is a self-assembling viscous solution that once applied, forms a transparent hydrogel 3-D matrix to promote local haemostasis. PuraBond® is a CE marked class III medical device approved in the European Union (EU) in 2014 including the United Kingdom (UK) as a member state at the time, which meets the General Safety and Performance Requirements (GSPR) of all relevant European Medical Device Regulations according to European Council Directive 93/42/EEC for medical devices and its relatives. The transparent nature of PuraBond® allows visualisation of the surgical field, permitting real-time assessment of intraoperative haemostasis [15]. PuraBond® has been shown to reduce the risk of delayed-onset haemorrhage following gastrointestinal endoscopic submucosal resection [16] and has also been utilised in cardiothoracic [17] and laparoscopic surgery [18]. In ENT, favourable outcomes have been reported in terms of reducing post-operative haemorrhage rates following turbinoplasty and after endoscopic endonasal surgery [19,20]. The cost of PuraBond® ranges between £200-£250 per syringe (3 ml) depending on the hospital procurement team contract with the manufacturer.

This study aimed to assess the utility of PuraBond® in patients undergoing TORS for the treatment of early-stage HPV-positive OPSCC. Given the haemostatic benefits of PuraBond® reported in the literature in other surgical specialties, the hypothesis was that it could also present a beneficial adjunct in TORS.

## Materials and methods

### Study design

This case series was performed at a single high-volume, tertiary referral, university hospital trust in the UK. The study period was October 2021–May 2022 and all consecutive patients who had suspected or confirmed oropharyngeal malignancy and underwent TORS with application of PuraBond® were included. Given that most complications from TORS (e.g. haemorrhage) occur within the first post-operative month, all patients were followed up for a minimum of 30 days. Patients were excluded if they had incorrectly coded notes or application of a different haemostatic agent other than (or in addition to) PuraBond® had been used. Case notes for all patients were analysed retrospectively. Data extracted included patient demographics (age, sex, smoking history), procedure details (date, indication for surgery and type of TORS and additional procedures performed) and tumour details (histopathology, including HPV status and stage).

The primary outcome measure was post-operative haemorrhage rate (primary; within 24 h of surgery, and secondary; between 1 and 30 days post-surgery). Secondary outcome measures included, length of hospital stay (LOS), post-operative swallowing complications, feeding tube insertion or tracheostomy either prior to or within 30 days of surgery, and hospital re-admission rate within 30 days of surgery. Surgeon-reported ease of PuraBond® application was also evaluated prospectively after each case. Patient details were pseudonymised, data extracted and uploaded to a digital spreadsheet. Descriptive statistical analysis was undertaken where applicable (mean, median, standard deviation) using Microsoft Excel® version 16.31 (Microsoft Inc., Redmond, WA). The study was registered in ClinicalTrials.gov Protocol Registration and Results System ID: NCT05405907 (https://register.clinicaltrials.gov). Ethical approval was not required as per the principles of the Declaration of Helsinki as this involved anonymous, retrospective analysis of case notes and investigations. This case series has been reported in line with the PROCESS Guideline [21].

### Surgical technique

**Fig. 1:**
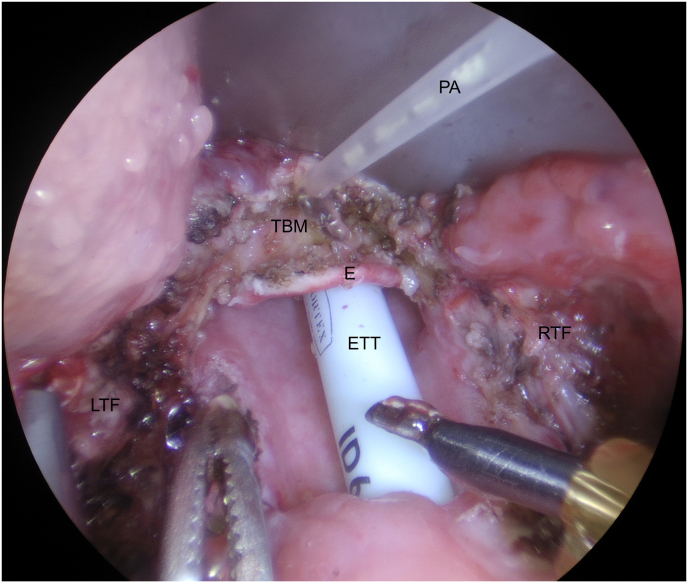
An endoscopic image following a diagnostic bilateral tonsillectomy and tongue base mucosectomy demonstrating application of Purabond® to the mucosectomy site. E = epiglottis, ETT = endotracheal tube, LTF = left tonsillar fossa, PA = Purabond® applicator, RTF = right tonsillar fossa, TBM = tongue base mucosectomy site.

All surgical cases were performed by the senior author, a consultant ENT – head and neck surgeon, with over six years of transoral robotic surgical experience, having independently performed over 60 TORS cases. All patients were optimised for surgery with pre-operative anaesthetic assessments and advised to stop relevant medications such as anticoagulants. The exact surgery performed was individualised to each patient according to their respective pathology. Use of the da Vinci® surgical robot (Intuitive Surgical Inc., Sunnyvale, CA) and set up were standardised across all cases. Standard set up involved spatula and Maryland instruments attached to each robotic arm, with monopolar (settings: 25W/25W) and bipolar (settings: 20W) cautery connected, respectively. All patients were anaesthetised with general anaesthesia and intubated via the nasotracheal route. Skin was prepared with Betadine solution and surgical drapes applied to create a sterile field. A 0-silk tacking suture was used for tongue retraction and positioning. The FK retractor and oral cavity spandex for cheek retraction were used to optimise transoral access. The indicated procedure was performed with one bedside surgeon assisting for suctioning, instrument changes, haemostasis with Ligaclips® (Ethicon Inc., Bridgewater, NJ) and application of PuraBond® among other activities. The primary (robotic) surgeon would sit remotely (within the same operating room) at the robotic console controlling the surgical instruments. The decision to apply PuraBond® was made prior to each procedure and applied to all consecutive TORS patients within the study inclusion period. Following completion of the resection and adequate haemostasis, 3 ml (one syringe) of PuraBond® were applied to the surgical bed of TORS resection (Fig. 1).

### Post-operative management

All patients were managed post-operatively according to the trust-specific TORS protocol. This included prescription of regular soluble analgesia (paracetamol, ibuprofen, gabapentin, codeine, and morphine) and laxatives for 10 days (all administered PO), as well as 24 h of tranexamic acid (1 g IV BD) and co-amoxiclav (1.2 g IV TDS). Oral intake was initially restricted to clear fluids following return to the ward from the operating room with patients commenced on a soft diet on day 1 post-operatively. Nasogastric (or other) feeding tube was not inserted routinely. Once adequate oral intake was resumed and analgesia optimised, patients were discharged with clear instructions about what symptoms to look for (bleeding, pain not controlled by the prescribed medication) and when to represent to the Accident & Emergency (A&E) department. Routine follow-up was arranged in 4 weeks in the ENT multidisciplinary team (MDT) clinic with Speech and Language Therapy (SLT) availability (if required) as face-to-face appointments in the outpatient setting.

## Results

Between October 2021 and May 2022, 13 consecutive TORS procedures were performed at the study centre, all of which included application of PuraBond® and therefore included in the study. These represented 12 patients as there was one second look and re-resection procedure. No patient was excluded and no patient was lost to follow-up. The cohort consisted of 6 male and 6 female patients. The mean age of the study population was 55 years with a range of 19–66 years. The patient group comprised of 7 ex-smokers (58.3%), 2 non-smokers (16.7%) and 3 current smokers (25.0%). Of the TORS procedures performed, 6 were diagnostic (46.2%) and 7 were therapeutic surgical interventions performed for early-stage neoplastic disease amenable to resection (53.8%). All diagnostic TORS procedures involved tongue base mucosectomy (TBM) (100%). Indications for TBM included mainly CUP (n = 5, 83.3%) and one mucosal tongue base lesion (n = 1, 16.7%). Therapeutic TORS procedures included lateral oropharyngectomy (n = 3, 42.9%), partial oropharyngeal resection (n = 3, 42.9%) and oropharyngeal re-resection (n = 1, 14.2%). Indications for therapeutic TORS included tonsillar squamous cell carcinoma (SCC) (n = 3, 42.8%), glossotonsillar sulcus SCC (n = 1, 14.3%), parapharyngeal space pleomorphic salivary adenoma (n = 1, 14.3%), paraganglioma (n = 1, 14.3%), and oropharyngeal re-resection for close (<1 mm) resection margins (n = 1, 14.3%). Analysis of tumour expression for p16 was relevant in 6 cases, in all of which, p16 was strongly expressed on immunohistochemistry (100%). There were 7 cases (53.8%) where additional procedure(s) were performed alongside the primary TORS procedure. A summary of the patient characteristics, surgical indications and procedure breakdown is presented in Table 1.

**Table 1:** Summary of the included patient characteristics, surgical indications, and procedure breakdown. CUP = carcinoma of unknown primary, ECA = external carotid artery, GORD = gastro-oesophageal reflux disease, SCC = squamous cell carcinoma, OPSCC = oropharyngeal squamous cell carcinoma.

Patient case	Age (years)	Sex	Co-morbidities	Smoking status	Indication for surgery	Surgical intent	TORS procedure	Additional procedure(s)	Histology & TNM stage	HPV status
1	60	Male	Nil	Smoker	OPSCC (left tonsil)	Therapeutic	Left partial oropharyngeal resection	Left selective neck dissection and left ECA ligation	SCC T2N2bM0	Positive
2	64	Male	Nil	Ex-smoker	OPSCC (right tonsil)	Therapeutic	Right partial oropharyngeal resection	Right neck dissection and right ECA ligation	SCC T2N2bM0	Positive
3	66	Male	Osteoarthritis	Ex-smoker	OPSCC (left tonsil)	Therapeutic	Left oropharyngectomy	Left modified radical neck dissection and left ECA ligation	SCC T2N2bM0	Positive
4	63	Male	Nil	Ex-smoker	OPSCC (left glossotonsillar sulcus)	Therapeutic	Left oropharyngectomy	Left neck dissection and left ECA ligation	SCC T1N2bM0	Positive
5	65	Female	Nil	Non-smoker	Parapharyngeal space tumour (right deep lobe parotid)	Therapeutic	Right parapharyngeal space tumour resection	Nil	Pleomorphic salivary adenoma (benign)	N/A
6	64	Male	Nil	Ex-smoker	Second look and re-resection procedure (case 2 – right tonsil)	Therapeutic	Right oropharyngectomy	Nil	SCC T2N2bM0	Positive
7	19	Female	Asthma	Smoker	Parapharyngeal space tumour (right cervical)	Therapeutic	Right cervical paraganglioma resection	Nil	Paraganglioma (benign)	N/A
8	38	Female	Goltz syndrome, hiatus hernia and osteoarthritis	Non-smoker	CUP (right cervical level II metastatic lymphadenopathy)	Diagnostic	TBM	Bilateral tonsillectomy	SCC T1N1M0	Positive
9	40	Female	Nil	Ex-smoker	Tongue base lesion (left)	Diagnostic	TBM	Nil	No evidence of primary tumour	N/A
10	63	Male	Nil	Ex-smoker	CUP (right cervical level II metastatic lymphadenopathy)	Diagnostic	TBM	Bilateral tonsillectomy	No evidence of primary tumour	N/A
11	55	Female	Nil	Ex-smoker	CUP (right cervical level II metastatic lymphadenopathy)	Diagnostic	TBM	Nil	No evidence of primary tumour	N/A
12	47	Male	Depression	Ex-smoker	CUP (right cervical level II metastatic lymphadenopathy)	Diagnostic	TBM	Nil	No evidence of primary tumour	N/A
13	65	Female	Mixed anxiety and depression and GORD	Smoker	CUP (right cervical level II metastatic lymphadenopathy)	Diagnostic	TBM	Nil	No evidence of primary tumour	N/A

In terms of the outcome measures, there were no patients who developed, either a primary or secondary, haemorrhage post-TORS (n = 0, 0%). There were also no re-admissions within 30 days of surgery (n = 0, 0%). Average LOS was 2.87 days (SD = 0.93 days) with a range of 1.48–4.54 days. In terms of swallowing outcomes, all patients resumed an oral diet on day 1 post-TORS which, facilitated by the early input of SLT Allied Health Professionals (AHPs), was rapidly built up to their normal oral intake prior to discharge. No patient required any type of feeding tube or a tracheostomy either prior to, during or within 30 days of TORS (n = 0, 0%). In all procedures, the feasibility of PuraBond® application was reported as ‘easy’ by the bedside surgeon (n = 13, 100%). These findings are summarised in Table 2.

**Table 2:** Summary of primary and secondary outcome measures. SD = standard deviation.

Outcome measure	n
Primary haemorrhage	0 (0%)
Secondary haemorrhage	0 (0%)
Requirement for feeding tube	0 (0%)
Requirement for tracheostomy	0 (0%)
Length of hospital stay (days)	0 (0%)
Hospital re-attendance or readmission	2.87 (SD = 0.93, range 1.48–4.54)
Reported ‘easy’ application of PuraBond®	13 (100%)

## Discussion

OPSCC rate is increasing rapidly worldwide due to the presence of HPV-mediated malignancies. This is estimated to account for more than 70% of cases across Europe [20] and carries a substantial burden in terms of morbidity, mortality, and quality of life (QoL). Recent advances in minimally invasive surgical techniques such as transoral laser microsurgery (TLM) and TORS have allowed surgery to become a viable primary treatment option in the management of these head and neck malignancies, who predominantly affect younger patients, naturally more likely to experience the long-term toxicity of (the alternative) CRT [22]. A systematic review and meta-analysis of more than 400 patients with OPSCC managed with TORS as the primary treatment showed promising oncological and QoL outcomes, comparable to those of CRT [23]. In addition, randomised controlled trials (RCTs) are currently being conducted aiming to risk-stratify early stage OPSCC patients and standardise treatment algorithms accordingly [24].

This case series looked at the utilisation of PuraBond® in ENT – head and neck surgery and is the first to specifically evaluate its role in TORS. The only other study in ENT relates to a case report of a 49-year-old patient, also with OPSCC, who underwent coblation for nasopharyngeal stenosis post-CRT where PuraBond® was shown to reduce the reformation of fibrosis two months after surgery [20]. Another recent study evaluated the role of PuraBond® in neck endocrine surgery, showing it to be both safe and efficacious in reducing the post-operative haemorrhage rate in open cervical surgery [25].

This case series illustrates that there may well be a role for PuraBond® in TORS too in view of the promising early findings, especially with regards to post-operative haemorrhage rates and swallowing outcomes, both Key Performance Indicators (KPIs) for TORS. The reported rates of post-operative haemorrhage following TORS vary in the literature, but typically range between 2.2 and 9.8% [12]. Thus, the observed rate in this case series appears favourable though larger prospective studies are needed.

In all OPSCC cases where TORS was employed for therapeutic purposes, HPV status was positive. It is widely reported that patients with HPV-mediated OPSCC are typically younger and generally fit at presentation [26]. QoL is therefore paramount in this patient cohort who will generally have longer to live with any treatment-related morbidity. Previous research has demonstrated that OPSCCC patients and their carers have reported dysphagia as the number one cause for distress and that this is an independent predictor for poor long-term QoL outcomes [27,28]. A meta-analysis in 2015 showed that 18–39% of TORS patients required a feeding tube post-operatively, with this becoming permanent in up to 7% of patients. The reported MD Anderson Dysphagia Inventory (MDADI) scores ranged from 65.2 to 78 [23]. A more recent prospective analysis of 25 patients undergoing TORS for OPSCC showed mean MDADI scores to be 74.5 with feeding tube requirements as high as 60% [29]. As such, the reported results in this case series also appear encouraging when it comes to swallowing outcomes post-TORS and would merit further dedicated studies on this crucial matter.

In terms of the secondary outcomes, the mean length of hospital stay was 2.87 days (range 1.54–4.31 days). This is a relatively short hospital stay for patients who have had primary oncological surgery [30]. This, in turn, is associated with patient benefits in terms of minimising hospital-associated complications such as nosocomial infection and venous thromboembolism. In addition, there are added cost benefits that come with a shorter post-operative inpatient stay. Reassuringly, there was also no suggestion that the patients evaluated were discharged prematurely, in view of no readmission(s) within 30 days post-TORS.

PuraBond® is part of the RADA16 product family and is a synthetic absorbable haemostatic agent. The favourable post-operative outcomes reported in this case series in relation to reduced post-TORS haemorrhage rates is of prime importance and merits further dedicated studies. This is because post-TORS haemorrhage can be catastrophic resulting in airway compromise and/or hypovolaemic shock, all of which can rapidly lead to death. Indeed, RCTs have had to be stopped early following deaths from haemorrhage in the TORS arm in view of the patient safety and ethical concerns [31]. The beneficial effect of PuraBond® in reducing postoperative haemorrhage rates has also been demonstrated in other interventions, most notably endoscopic resection of gastrointestinal mucosal lesions [16]. It has also been used effectively in managing active bleeding in cardiothoracic [17], gastrointestinal [32,33], and acute settings [34].

When it comes to swallowing and feeding tube outcomes, the reported findings support a possible beneficial effect associated with PuraBond®. This beneficial effect has been postulated to be likely due to the synthetic mesh-like interwoven fibres of the RADA16 gel acting as a template for tissue repair following surgery [15]. In *in vitro* models, the application of RADA16 has been shown to support proliferation of different cell types [35], and faster re-epithelisation of the collagen matrix [36]. Animal models have also shown similar results in terms of facilitating wound healing and tissue regeneration [37,38]. Once again, the reported early findings in this study would support the need for further research including with more robust comparative trials evaluating the role of PuraBond® in TORS and its impact on functional outcomes.

Despite these preliminary promising findings, it is paramount to also consider the limitations of this study. The most important limitation relates to its small cohort (n = 13). Moreover, the study design was retrospective and there was no control group. The study was also limited through its assessment of complications up to 30 days post-operatively. Although most TORS complications occur within this timeframe, it is possible that patients may present with secondary haemorrhage beyond 30 days. Finally, no objective or quantitative measures (via validated tools such as MDADI) were used to assess swallowing. The above limitations naturally limit the scope of the conclusions that can be derived. However, the primary aim of this case series was to act as a preliminary ‘proof-of-concept’ study regarding the potential role of PuraBond® in TORS in view of its proven benefits in other settings, so as to guide further research on the subject. In that respect, this study appears to have achieved its goal.

## Conclusion

This case series is the first to evaluate the role of PuraBond® in TORS for oropharyngeal cancer. The reported favourable early outcomes, spanning across a range of key domains, including post-operative haemorrhage, swallowing, pain, and length of hospital stay, albeit preliminary, support its safety and feasibility in TORS and can serve as a platform to help direct future research directions on the subject. The current early findings support the need for larger, prospective, controlled (and ideally randomised) studies with longer follow-up, to better define whether the known haemostatic and regenerative properties of PuraBond® translate into direct patient benefit in the rapidly expanding field of TORS.

## Ethical approval

Ethical approval was not required as per the principles of the Declaration of Helsinki as this was an anonymous, retrospective analysis of case notes and investigations.

## Sources of funding

No funding was received for the production of this work or preparation of this manuscript. 3D Matrix Ltd who manufacture Purabond® paid for the open access fees but had no role in the study design, data collection and analysis, decision to publish, or preparation of the manuscript.

## Author contribution

**Keshav Kumar Gupta:** conceptualisation, methodology, formal analysis, writing – original draft, writing – review and editing. **Georgios Garas:** conceptualisation, methodology, writing-review and editing. **Matthew Idle:** conceptualisation, methodology, writing-review and editing. **Susan Germain:** methodology, writing-review and editing. **Mriganka De**: conceptualisation, methodology, supervision, writing – review and editing.

## Consent

Patient consent was not required as this was an anonymous, retrospective analysis of case notes and investigations.

## Registration of Research Studies

1. Name of the registry: ClinicalTrials.gov.

2. Unique Identifying number or registration ID: NCT05405907.

3. Hyperlink to your specific registration (must be publicly accessible and will be checked): https://clinicaltrials.gov/show/NCT05405907.

## Guarantor

Mriganka De.

## Provenance and peer review

Not commissioned, externally peer-reviewed.

## Declaration of competing interest

1) Mriganka De is an employed consultant for 3D Matrix Ltd who manufacture Purabond®

2) All other authors have no conflicts of interest to declare. Georgios Garas is Consultant ENT, Head and Neck Surgeon (Surgical Oncology) at University Hospitals Birmingham NHS Foundation Trust (Birmingham, UK) and Honorary Clinical Senior Lecturer (Surgery and Cancer) at Imperial College London (London, UK). There are no commercial interests to declare.
